# Exercise-Associated Hyponatremia: 2017 Update

**DOI:** 10.3389/fmed.2017.00021

**Published:** 2017-03-03

**Authors:** Tamara Hew-Butler, Valentina Loi, Antonello Pani, Mitchell H. Rosner

**Affiliations:** ^1^Oakland University School of Health Sciences, Rochester, MI, USA; ^2^SC Nephrology and Dialysis, Brotzu Hospital, Cagliari, Italy; ^3^Department of Medicine, University of Virginia Health System, Charlottesville, VA, USA

**Keywords:** exercise, hyponatremia, rhabdomyolysis, water, pathogenesis

## Abstract

Exercise-associated hyponatremia (EAH) was initially described in the 1980s in endurance athletes, and work done since then has conclusively identified that overdrinking beyond thirst and non-osmotic arginine vasopressin release are the most common etiologic factors. In recent years, EAH has been described in a broader variety of athletic events and also has been linked to the development of rhabdomyolysis. The potential role of volume and sodium depletion in a subset of athletes has also been described. This review focuses on the most recent literature in the field of EAH and summarizes key new findings in the epidemiology, pathophysiology, treatment, and prevention of this condition.

## Introduction

Exercise-associated hyponatremia (EAH) refers to a low blood sodium concentration ([Na^+^]) that develops during or immediately following physical activity (sport or recreation). For most labs, the diagnostic threshold for hyponatremia is any blood [Na^+^] below135 mmol/L regardless of the presence or absence of signs and symptoms.

Isolated cases of severe, clinically significant, EAH with associated encephalopathy were first reported in the 1980s in ultramarathon (>42 km) runners (1, 2). Ironman triathletes—particularly those competing in hot climates—were also finishing races with mild EAH (3). Since then, EAH cases have been reported outside of prolonged endurance exercise and in (otherwise healthy) individuals participating in team sports (4, 5), shorter races (6), and yoga classes (7, 8). Unfortunately, despite increased recognition and research performed on EAH worldwide (9), athletes continue to die from complications associated with hyponatremic encephalopathy (10–14).

The purpose of this review is to provide an update on EAH. Sports medicine’s evolving understanding of EAH appears to more closely align with the clinical spectrum and pathological manifestations of hyponatremia seen in outpatient and inpatient care settings. We aim to highlight new information with regards to the epidemiology, pathophysiology, treatment, and prevention of EAH and not a comprehensive overview, which is available elsewhere (9, 15–17).

## Epidemiology of EAH

Once a rare and isolated phenomenon limited to ultrarunners (1, 2), hikers (18, 19), and Ironman triathletes (3, 20, 21), EAH awareness blossomed in the early 2000s when hyponatremia began surfacing in marathon runners (22–26). The tragic deaths of two female charity marathon runners in 2003 (25) fully exposed this fatal complication of exercise to sports medicine personnel as well as the lay public. Scientific interest piqued in parallel with growing EAH incidence rates around the world (27–37). Collective studies demonstrated that overzealous fluid consumption (above the dictates of thirst) coupled with fluid retention [exercise-induced non-osmotic arginine vasopressin (AVP) secretion] was the primary cause of EAH in athletes participating in prolonged endurance exercise (9, 25).

It has been hypothesized that mentruant females are at increased risk for developing EAH compared with males (9, 26, 38), due to estrogen-mediated impairment of cerebral adaptation to rapid osmotic swelling (39). However, males are not immune toward developing asymptomatic (5) or severe symptomatic EAH with fatal consequences (10–13, 32, 40–43). Although the majority of EAH cases are documented in females, a large study performed on Boston Marathon runners suggested that the apparent sex difference disappeared when data were adjusted for body mass index and racing times (29).

Over the past decade, EAH deaths have been confirmed in the lay press in high school football players following practice (10–12), a soldier on the first day of Ranger training (44), a policeman participating in a 19 km bike ride (45), a college student performing calisthenics for a fraternity (42), a bushwalker (43), an ironman triathlete (14), and a canoeist during an ultradistance race (13). Additionally, a highly fit solider died during a 50 km training march with both hyponatremic encephalopathy and exertional heat stroke (41). The literature also reports symptomatic cases of EAH after long distance swimming (46), mountain cycling (47), yoga (7, 8), 2 h of weightlifting plus tennis (48), and in an individual with cystic fibrosis after low-intensity lawn bowling (49).

Cases of asymptomatic EAH (diagnosed through routine screenings for research purposes) have recently been documented in 33% of 10 rugby players following an 80-min match (4), 70% of 30 elite junior rowers during an extended training period (5), 11% of 1,089 Ironman triathletes tested post-race (50), 6% of 33 endurance cyclists tested pre- and post-race (51), 67% of 15 ultramarathon runners testing during the race (52), 5% of 161 marathon and half-marathon runners tested pre-race, and 8% of 192 marathon and half-marathon runners tested post-race (6). Thus despite increased awareness of the hazards of overdrinking, EAH fatalities, case reports, and incidence rates have spread into a wider variety of sporting activities.

**Figure 1:**
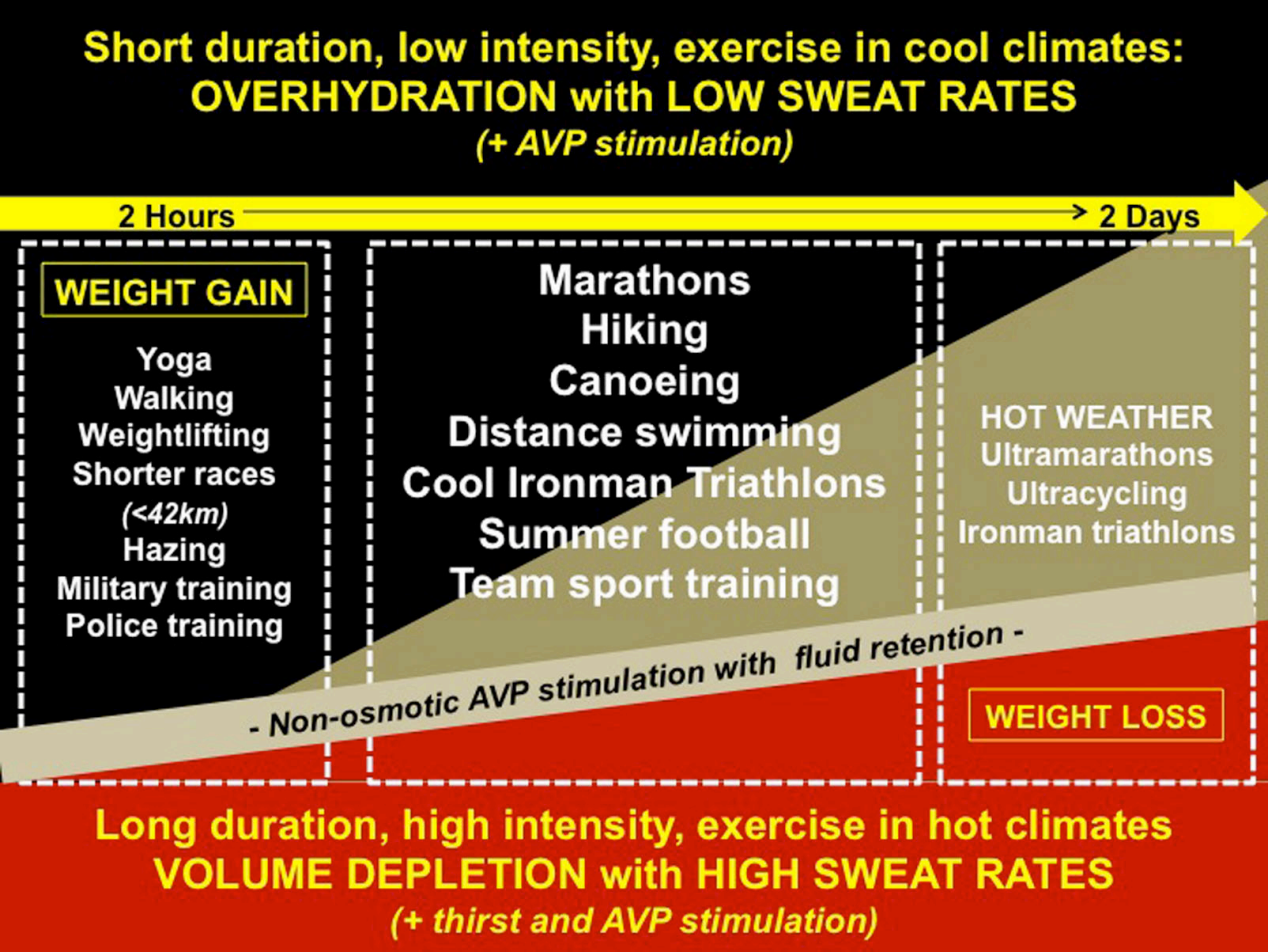
**Reported cases of exercise-associated hyponatremia (EAH) along the spectrum of pathophysiology**. Cases of EAH per sport are superimposed upon the broad spectrum of volemic pathophysiology: (1) hypervolemia—overzealous drinking with arginine vasopressin (AVP) stimulated fluid retention and minimal sodium losses or sodium over-correction (black triangle); (2) euvolemia—moderate drinking with AVP stimulated fluid retention and mild (compensable) sodium losses (gold triangle); and (3) hypovolemia—moderate drinking (volemic thirst) with AVP stimulated fluid retention, and under-replaced sodium losses (red triangle).

Figure 1 summarizes recent reported cases of EAH, referenced above, within a wide variety of sporting and recreational activities. These cases are superimposed upon the broad spectrum of pathophysiological volume states associated with hyponatremia ranging from hypervolemia to hypovolemia. Of note, exercise-induced non-osmotic AVP secretion (with persistent fluid retention) permeates throughout the entire pathophysiological spectrum. A comprehensive reference list of all EAH cases is provided in a recent review (9).

## Pathogenesis of EAH

**Figure 2:**
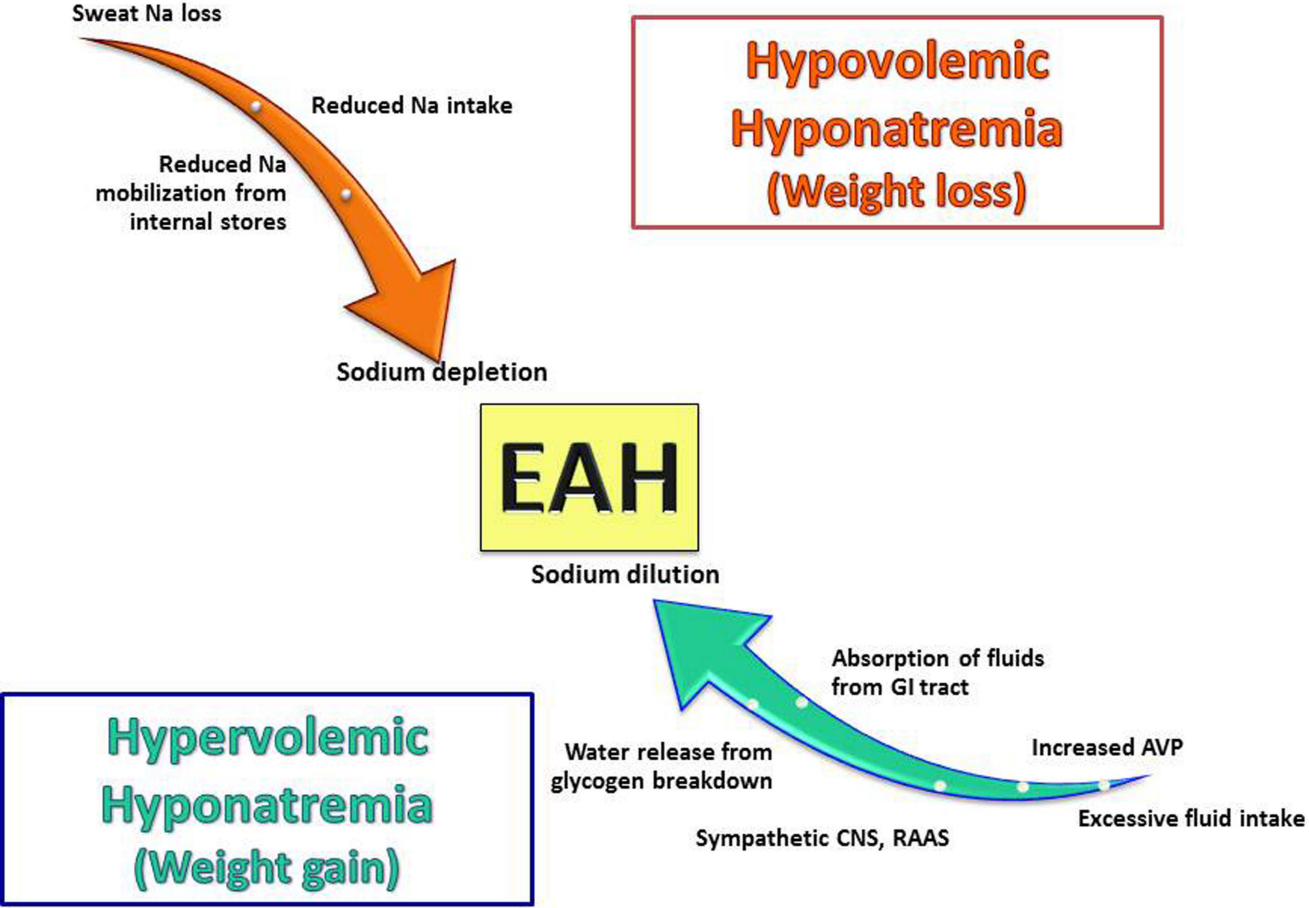
**Pathogenic factors involved in the development of exercise-associated hyponatremia (EAH)**. EAH develops from multiple mechanisms and can occur in both states of volume excess and depletion with AVP secretion occurring in both cases. It is important to note that none of these mechanisms occur in isolation, and they can overlap in any given patients (for example, sodium loss and excess water intake can occur in the same patient) Abbreviations: Na, sodium; AVP, arginine vasopressin; GI, gastrointestinal; CNS, central nervous system; RAAS, renin–angiotensin–aldosterone system.

Exercise-associated hyponatremia has a complex pathogenesis with multiple overlapping etiologies (15). In 2015, the Third International Exercise-Associated Hyponatremia Consensus Development Conference concluded that the primary etiology and mechanism leading to EAH was the overconsumption of hypotonic fluids likely in combination with non-osmotic stimulation of AVP secretion (9). However, recent data support other mechanisms that may be operable with variable contributions to the overall development of hyponatremia. This section reviews both known as well as more novel mechanistic features (Figure 2).

### Overconsumption of Hypotonic Fluids

The majority of athletes who develop hyponatremia demonstrate an increase in total body water (TBW) relative to that of total body exchangeable sodium (15, 29, 30). This develops from by the ingestion of hypotonic fluids (water or sports drinks) in excess of sweat, urine, and insensible (mainly respiratory and gastrointestinal) losses (i.e., drinking in excess). It is likely that conditioned behavior as well as inappropriate hydration recommendations (that are often taken to the extreme such as recommendations to “drink as much as possible”) account for this excessive fluid intake (9, 22). This is compounded by the wide availability of fluids along event courses.

The data supporting overhydration as the major mechanism involved in EAH is derived from the observations of weight gains seen in the majority of, but not all, athletes who become symptomatic with EAH (30). These rapid weight gains can only occur through ingestion of fluids in excess of body losses. As an example of the magnitude of these fluid intakes, individuals with normal glomerular filtration rates who are ingesting a Western diet can excrete between 500 and 1,000 mL/h of water. When non-renal losses of water (such as sweat and insensible fluid losses) are included into the net fluid balance equation, individuals can excrete 1,000–1,500 mL/h of dilute urine before developing water retention (15). While some athletes may drink this much, many do not and thus additional factors must be involved in the development of EAH.

### Non-Osmotic AVP Secretion

Arginine vasopressin is the major hormonal regulator of water excretion, and abnormalities in AVP secretion are likely operative in the majority of athletes with EAH (25, 53–56). Under normal circumstances, AVP is suppressed in the presence of hypoosmolality. In some athletes, AVP is not appropriately suppressed (as typified by the finding of inappropriately elevated urine osmolality) (25, 52–56). This release of AVP leads to water retention in the distal tubule of the kidney and impaired water excretion. Coupled with excessive water intake, inappropriate water retention will lead to hyponatremia. Several potential stimuli to AVP release in exercising athletes include non-specific stresses (pain, emotion, and physical exercise), nausea/vomiting, hypoglycemia, exposure to heat, and medication use (such as non-steroidal anti-inflammatory drugs and selective serotonin reuptake inhibitors) (57–61).

A more speculative mechanism is that AVP may be stimulated by the release of inflammatory cytokines (25). In particular, interleukin-6 (IL-6) has been shown to increase during exercise by both physiologic (as it is involved in the mobilization of energy stores during exercise) and pathologic means (with muscle inflammation from breakdown) and may serve as a stimulus for AVP production. A study on 15 participants in ultramarathons, found a significant positive correlation between AVP and IL-6 (*r* = 0.31, *P* < 0.05) but not between AVP and blood glucose or ambient temperature (52).

While AVP is secreted in response to volume depletion, the typical levels of volume depletion in athletes are less than 7–10%; the threshold for AVP release. However, a study on rowers during a 4-week training camp revealed an inappropriate lack of AVP suppression occurring with EAH (plasma copeptin levels were used as a surrogate marker of AVP). In this case, the potential non-osmotic stimuli to AVP were thought to be related to hypovolemia as reflected by an association with increased hematocrits (5). Thus, the role of hypovolemia as stimulus for EAH may be underappreciated.

### Sweat Sodium Loss

The issue as to the contribution of sweat sodium loss to the development of EAH remains controversial. Sodium loss from sweat is highly variable between individuals, and compared with the general population, endurance athletes have lower sweat sodium levels (62, 63). The loss of hypotonic sweat would be expected to raise the serum sodium. However, sweat loss could contribute to the development of hyponatremia in two manners: (1) if the degree of fluid loss was sufficient to produce significant volume depletion and provides a stimulus to AVP release, thereby impairing excretion of water and/or (2) through ingestion of fluids that were more hypotonic than the fluid losses. This scenario may contribute to the finding of EAH developing in some athletes with net weight loss.

### Sodium Mobilization from Internal Non-Osmotically Active Stores

A large amount of the total body sodium may exist in bone, skin, and cartilage stores that are not osmotically active but potentially recruitable into an osmotically active form (64). This may be as large as one fourth of the total sodium in the body. These sodium stores are reversibly bound to substances such as glycosaminoglycans and may be “dynamic,” meaning that that sodium can enter and exit this pool (65). Sodium exiting this pool may buffer the serum sodium and lower the risk of hyponatremia. On the other hand, sodium entering this pool may exacerbate hyponatremia (66). It has been hypothesized that athletes who develop EAH either cannot activate the exchangeable pool of sodium in response to sodium losses or alternatively sodium may move into non-osmotically active forms (30). The mechanisms that control the exchange of sodium between these compartments are unknown, and this mechanism remains speculative. However, these exchangeable sodium stores may be the explanation why some athletes gain significant weight yet do not become hyponatremic.

### Other Factors

In addition to the excessive intake of water, glycogen metabolism may be an important component in the cause of hyponatremia that occurs without weight gain because each kilogram of glycogen can contain upwards of 3 kg of associated metabolic water (15). As glycogen is metabolized, water is released and if not excreted could lead to depression of the serum sodium.

As renal water excretion is dependent upon the delivery of filtrate to the diluting segments of the distal nephron, the neurohormonal activation of the sympathetic nervous system and renin–angiotensin–aldosterone system that occurs with endurance exercise may be contributory and permissive (15). This is due to the fact that catecholamines and angiotensin II increase sodium and water reabsorption in the proximal tubule. This means that there will be decreased filtrate that is delivered to the distal diluting segments of the kidney, eventually leading to impairments of free water excretion.

Another contributing factor at the end of an event may be rapid absorption of fluids from the gastrointestinal tract. In athletes who may have a large amount of fluid in the stomach (from recent ingestion) and have elevated AVP levels, rapid absorption of this fluid (as gastrointestinal blood flow post-event increases) along with impaired free water excretion may set up athletes to develop hyponatremia (15).

The role of sodium ingestion during events remains a matter of debate, and there are little data supporting the concept that inadequate sodium intake either prior or during an event contributes to the development of EAH. Hoffman et al. found that hyponatremia, as well as exercise-associated muscle cramping, dehydration, and nausea or vomiting, was unrelated to total sodium intake in participants of a 161 km ultramarathon (67, 68). This was confirmed in a case study which demonstrated that oral sodium supplementation does not necessarily prevent symptomatic EAH associated with overhydration (69). On the other hand, another study demonstrated that longer term (10 days) reduction in dietary sodium intake can cause reductions in plasma sodium concentration before as well as during exercise when fluid losses and ingestion are large and may consequently have adverse effects on physiological and functioning during such exercise (70).

### Hypovolemic EAH

There has been debate in the literature as to the prevalence and role of hypovolemia in the pathogenesis of EAH. Earlier data supported the fact that the vast majority of EAH cases were in athletes with net weight gain and hypervolemia. However, in the past few years, more data have supported that hypovolemia is more prevalent than previously thought. In a recent Czech study on ultra-endurance races, 85% of the hyponatremic athletes were volume depleted based on body weight changes (71). Weight loss in EAH cases supports volume depletion as a contributor to EAH perhaps through non-osmotic AVP secretion as described above coupled with sodium losses.

## Newer Concepts in EAH: Rhabdomyolysis

**Figure 3:**
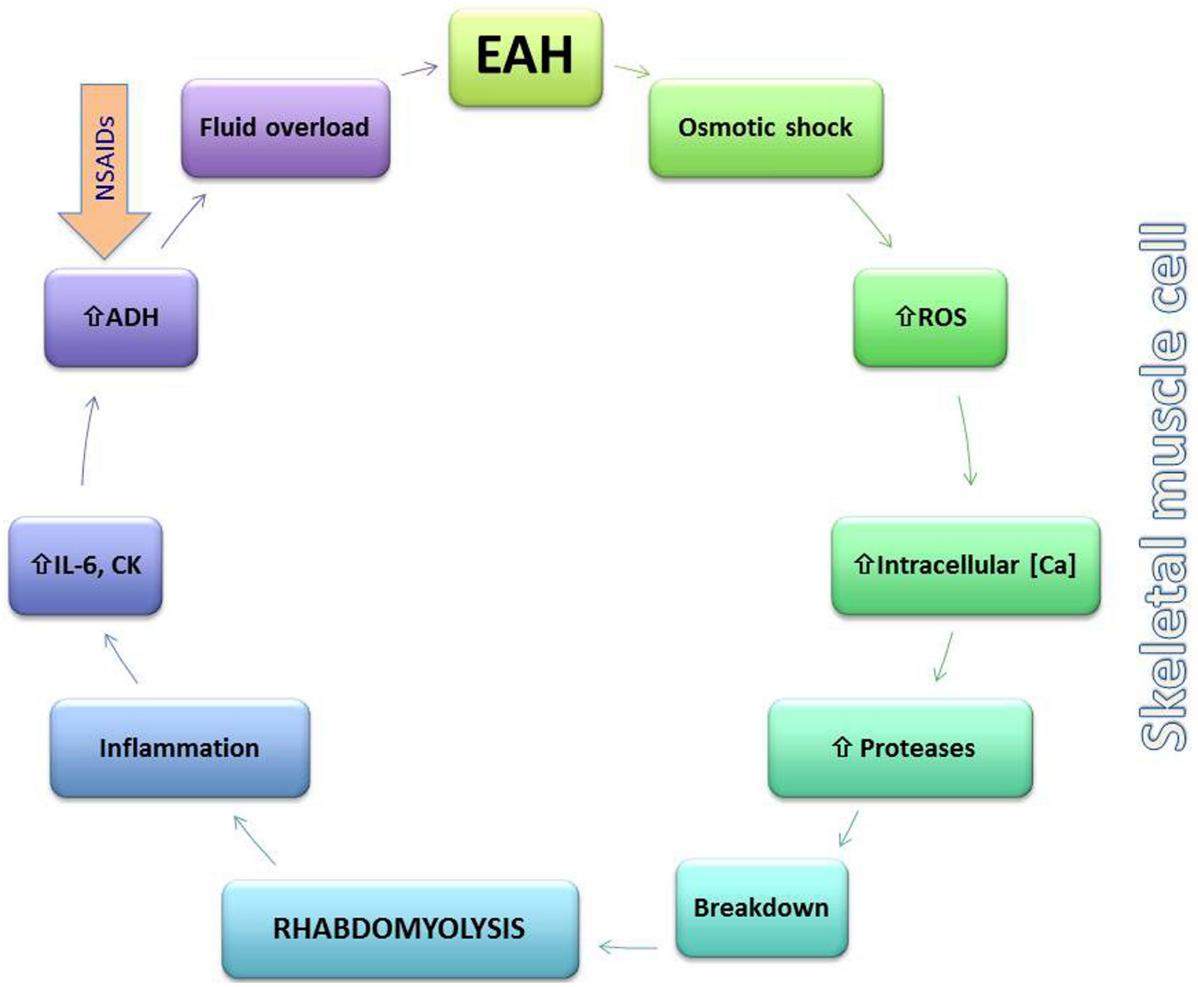
**Putative bidirectional relationship between exercise-associated hyponatremia (EAH) and rhabdomyolysis**. EAH may lead to rhabdomyolysis through changes in the skeletal muscle membrane (osmotic shock) and activation of reactive oxygen species (ROS) along with increases in intracellular calcium (Ca) that activate intracellular proteases and lead to cellular breakdown. Rhabdomyolysis either through this mechanism or by other means leads to local and systemic inflammation and release of interleukin-6 (IL-6), which may increase arginine vasopressin (AVP) levels further lowering serum sodium. Non-steroidal anti-inflammatory drug use may also contribute to excess AVP release.

A bidirectional association between rhabdomyolysis and EAH has been suggested in various studies and case reports (Figure 3) (72–74). Based on data from seven different ultra-endurance races and disciplines, Chìbkovà et al. reported that hyponatremic athletes tended to develop exercise-induced rhabdomyolysis more frequently than normonatremic ones (75). Cairns and Hew-Butler observed that transient hyponatremia preceded significantly higher elevation in creatine kinase (CK) during a marathon footrace (76). Rhabdomyolysis may be both an etiological factor in the development of EAH as well as sequelae of its development. Rhabdomyolysis link to the development of EAH may be through stimulating AVP secretion, possibly through increases of IL-6 and CK (25). On the other hand, EAH may cause rhabdomyolysis through changes in intracellular potassium and/or calcium concentrations, which reduce cell membrane stability and cause muscle cell injury (72). Interestingly, it is the return to normonatremia (cell volume decrease following a hypoosmotic increase), which appears to initiate the pathological intracellular calcium handling and consequent skeletal muscle injury (76).

## Treatment of EAH

**Figure 4:**
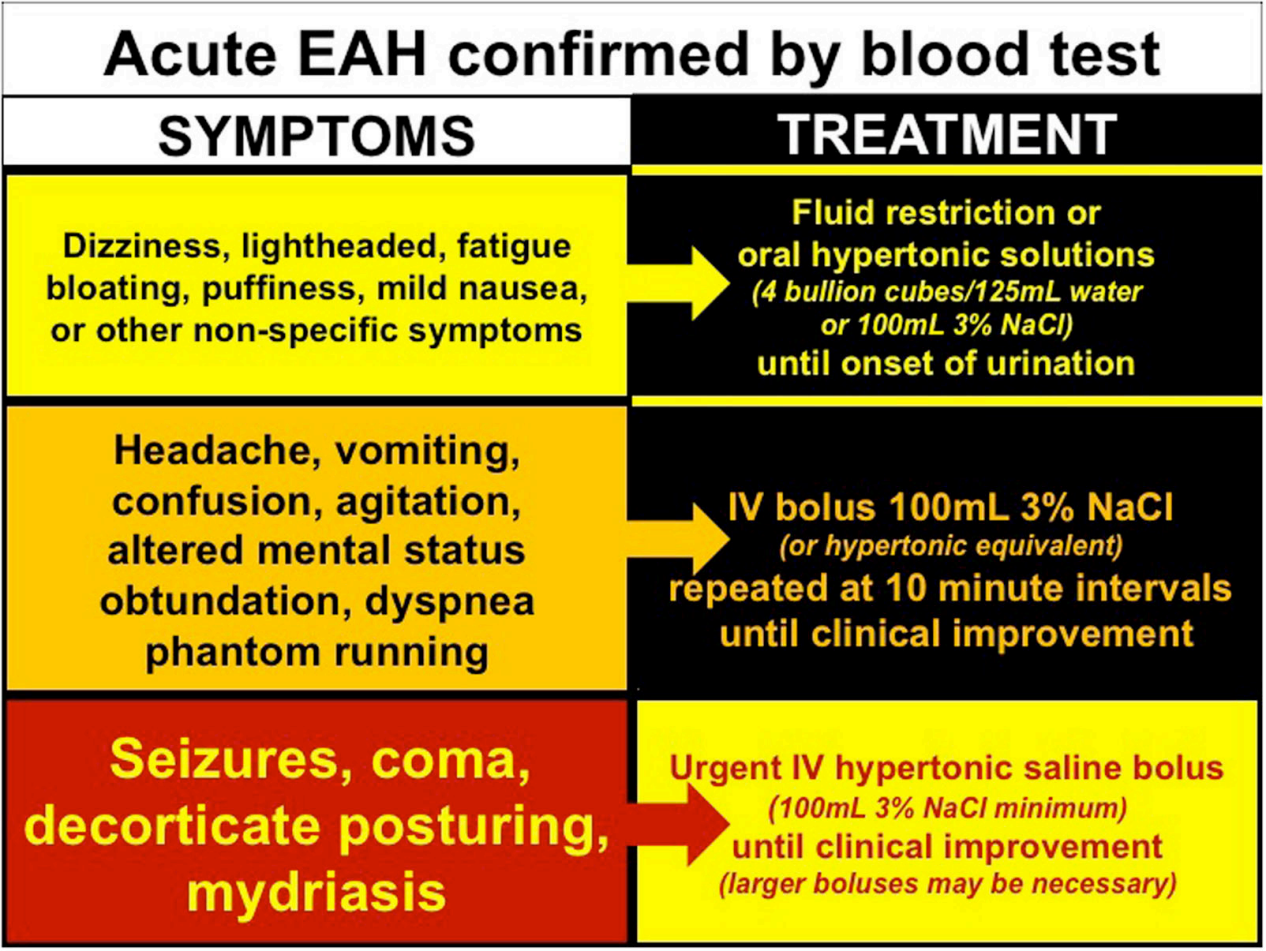
**Treatment of exercise-associated hyponatremia (EAH) according to signs and symptoms**. Evidenced-base treatment options for EAH associated with mild (yellow), moderate (orange), or severe clinical signs and symptoms.

Exercise-associated hyponatremia is largely an acute hyponatremia, clinically defined as having an onset within 48 h (77). It is unclear whether or not multistage races or frequent training bouts (over days to weeks) induce cerebral adaptations (i.e., extrusion of organic osmolytes such as glutamate, taurine, and myo-inositol), which would characterize the less symptomatic chronic form of hyponatremia (78). The ability of cerebral neurons to resist osmotic swelling, *via* adaptive processes undertaken by surrounding astrocytes over 1–2 days, significantly affects both symptoms and treatment (78). Without data to support the chronic variant of EAH, however, we will discuss the treatment of EAH as an acute variant. Accordingly, the treatment of EAH is guided by clinical signs and symptoms (Figure 4).

Collective evidence from case studies (25, 26, 79–82) and case-controlled trials (22, 83, 84) confirm that intravenous (IV) administration of a hypertonic saline solution is the most effective treatment for reversing encephalopathy associated with symptomatic EAH while increasing blood [Na^+^] (84). To date, there have been no complications associated with this life-saving treatment despite concerns of central pontine myelinolysis (85) or pain and/or scarring from administration into a peripheral vein (86). The recommended hypertonic bolus is 100 mL of 3% saline (or closest equivalent) administered every 10 min until resolution of encephalopathy signs and symptoms (9). When EAH encephalopathy is severe, hypertonic saline may be given in larger doses and at more frequent intervals, with as much as 950 mL of 3% saline needed to successfully reverse EAH encephalopathy in one case (87) and 40 mL of 20% saline used in another (46).

New evidence suggests that oral hypertonic saline solutions may reverse symptomatology (88) and reverse blood [Na^+^] (83, 84), when tolerated. Although these studies are preliminary, oral hypertonic solutions appears to be a more expedient option to classical treatment with fluid restriction. Exercisers with documented (biochemical) hyponatremia but with mild to moderate symptoms have responded favorably to small boluses of hypertonic solutions as either concentrated chicken broth (four bouillon cubes in 125 ml/one-quarter cup of water) (88) or 100 mL of 3% saline flavored with Crystal Light™ (83, 84). Although hypothesized, the efficacy of urea (89), mannitol, and/or AVP antagonists has yet to be tested as viable treatment options for EAH.

Isotonic (0.9%) saline has been successfully utilized in the treatment of EAH (1, 2, 80). However, the clinical and biochemical response is quite delayed when compared with hypertonic saline administration (days versus hours) (22, 80). The primary clinical concern with IV isotonic saline administration is exacerbation of fluid retention coupled with urinary sodium excretion (90) in the likelihood that exercise-associated non-osmotic AVP secretion is present (91). The *only* rare (but notable) exception is when EAH is associated with signs and symptoms of volume depletion, as noted above (i.e., elevated blood urea nitrogen, weight loss, and scant urine with urine [Na^+^] <30 mmol/L, thirst). In these rare pathophysiological exceptions (i.e., after prolonged exercise in hot conditions in unacclimitized individuals or in individuals with chronically elevated sweat or urinary sodium content), plasma volume expansion with both sodium and water (isotonic saline) may be preferred over administration of concentrated sodium solutions (hypertonic saline) (9, 77, 92). Serial monitoring of blood [Na^+^] should be performed regardless of treatment. When in doubt, hypertonic saline is an effective treatment option regardless of volemic status. Hypotonic solutions are absolutely contraindicated when a diagnosis of EAH is confirmed.

## Prevention of EAH

Prevention of EAH is of critical importance and requires organized educational programs with information disseminated to coaches, athletes and event staff regarding healthy hydration practices, sodium supplementation, and recognition and treatment of EAH. All of the deaths attributable to EAH would have likely been prevented if individuals had a better understanding of hydration needs as well as being able to rapidly recognize the signs and symptoms of EAH.

### Education Programs

There are many misconceptions regarding hydration needs during exercise that foster the belief that athletes and individuals performing even moderate levels of exercise should “drink as much fluid as possible” (93). Unfortunately, there are also various contests and challenges that are prevalent on the Internet that urge participants to drink as much water as possible in short periods of time, often with fatal consequences (94). Hoffman et al. assessed the quality of information currently available to the public on the Internet regarding hydration recommendations during exercise (95). Not surprisingly, the quality of information was poor even for those sites that would be considered “medical” or “scientific.” For example, only 7.3% of 110 web sites discussed that fluid intake should be based upon thirst, and the potential risk of hyponatremia from overhydration was noted by less than half the websites (95). Dissemination of more appropriate hydration guidelines is critical, and we recommend that education programs stress the following concepts that are evidence based:

1. Excessive fluid replacement that goes beyond thirst has not been shown to decrease the development of fatigue, muscle cramping or exertional heat stroke (9).
2. Modest levels of dehydration are tolerable and pose little risk in healthy individuals. Studies indicate that fluid deficits less than and up to a volume approximately equal to 3% of normal body mass (or ~5% TBW) can be well tolerated (96).
3. Utilize strategies during exercise to prevent overhydration.

- *Drink according to thirst*. Because fluid losses through sweat and urine are highly dynamic and variable across individuals participating in a variety of outdoor activities, recommending fixed ranges of fluid intake is not appropriate. The most individualized hydration strategy before, during, and immediately following exercise is to drink fluids when thirsty. Following thirst as a real-time guide appears safe and effective (9, 97).
- *Reduce the availability of fluids along the routes of exercise*. This strategy has been shown to reduce the incidence of hyponatremia during endurance events (98, 99). However, further studies are needed in order to determine the optimal number and spacing of fluid stations in various environment conditions.
- *Use monitoring of weight changes during exercise*. Body weight is a reasonable, even if not precise, surrogate measure of hydration state, and can be used to assess changes in hydration state (9). Even if body weight changes are imprecise during longer duration exercise it will be the expectation is that athletes should not gain weight during the event. Weight gain is an indication that fluid intake has been in excess of fluid losses, and water overload is present to some degree (9). Therefore, those individuals with weight gain should reduce their fluid intake. Whenever possible, access to scales and education on personal weight changes that occur with exercise should be available. It is important to note that in some particular environments EAH has been reported with substantial weight loss, therefore weight loss cannot always be considered a reliable approach for excluding the diagnosis of EAH.

Finally, we strongly recommend “grass-roots” efforts to adopt educational strategies to improve knowledge of safe hydration practices. These efforts should target a broad audience but must focus on those individuals such as coaches, event support staff and medical personnel that are most likely to have the greatest influence on the behavior of athletes. There is evidence that such strategies do work to lower the incidence of EAH when implemented (99–102). Furthermore, education programs should include recognition of the signs and symptoms of EAH as well as the need for immediate medical attention.

### The Role of Sodium Supplementation

While there is common consensus that the primary strategy to prevent EAH is to avoid drinking excessive fluids, the role of sodium supplements in decreasing the risk of EAH remains controversial (68). Previous work on the influence of sodium supplementation on plasma sodium levels has shown variable results. Vrijens and Rehrer showed that fluid replacement with a sports drink containing 18 mmol/L of sodium as compared with water, led to an attenuated fall in plasma sodium during 3 h of cycling in the heat (103). Anastasiou et al. showed that even small amounts of sodium ingested during 3 h of racing in the heat were sufficient to attenuate the decrease in plasma sodium (104). Similar findings were observed by Twerenbold et al. during a 4 h running time trial in temperatures ranging from 5 to 19°C. Again, sodium ingestion resulted in a smaller decrease in the plasma sodium concentration from pre- to post-run measurements in female athletes (105). Conversely, Barr et al. reported no differences in the plasma sodium concentration at the end of 6 h of ergometer use in the heat, when either a water or saline solution was ingested (106).

More recent data have led to continued controversies regarding the value of sodium supplementation. A case report has been recently published in which a runner with a prior history of EAH consulted a sports nutritionist who advised him to consume considerable amounts of supplemental sodium, which did not prevent him from developing symptomatic EAH during a subsequent long run (69). A study involving 156 participants of a 161 km race found a weakly positive relationship between sodium supplementation and post-race serum sodium concentrations; the authors concluded that sodium supplementation had a minimal contribution on the prevention of hyponatremia (68). In nine endurance-trained men, each following a low- or high-sodium diet for 9 days, Koenders et al. found that, despite decreased urinary sodium losses, plasma sodium was lower in the patients on low-sodium diet before and throughout exercise. They concluded that the general population recommendations to lower salt intake may not be appropriate for endurance athletes, particularly those training in the heat that may be at risk for EAH (70).

Turner et al. examined the novel hypothesis that sodium excreted in sweat during physical activity offsets a significant fraction of excess dietary sodium intake, and hence may contribute part of the health benefits of exercise. Hence, replacing sodium lost in sweat during exercise may improve physical performance and slightly lower the risk of EAH, but may attenuate the long-term health benefits of exercise (107).

In conclusion, as reported on the Statement of the 3rd International Exercise-Associated Hyponatremia Consensus Development Conference, while sodium ingestion during a race may attenuate the fall in blood sodium concentrations, it cannot prevent EAH in the setting of excessive fluid intake (94). It is the amount of fluid ingested rather than the amount of sodium ingested during exercise that drives the final blood sodium concentrations. Sodium-containing sports drinks, which are hypotonic, will not prevent EAH in athletes who overdrink during exercise.

## Summary

The incidence of EAH continues to spread into a wider variety of sporting activities and cause unnecessary deaths in otherwise healthy individuals. Drinking beyond thirst continues to be the primary pathophysiological factor in the development of EAH, regardless of mode of physical activity. Administration of hypertonic saline continues to be lifesaving, with treatment largely guided by clinical signs and symptoms. For mild to moderate EAH (without altered mental status), fluid restriction or oral hypertonic saline is recommended while for severe symptomatic EAH (with altered mental status), urgent administration of IV boluses of 100 mL 3% NaCl is required. Drinking according to the dictates of thirst, during and immediately following exercise will prevent the development of EAH when exercise is performed in temperate climates with duration of less than 17 h.

## Author Contributions

All of the authors contributed equally to the preparation and editing of this paper.

## Conflict of Interest Statement

The authors declare that the research was conducted in the absence of any commercial or financial relationships that could be construed as a potential conflict of interest.
